# Measurement Noise Covariance-Adapting Kalman Filters for Varying Sensor Noise Situations

**DOI:** 10.3390/s21248304

**Published:** 2021-12-12

**Authors:** Anirudh Chhabra, Jashwanth Rao Venepally, Donghoon Kim

**Affiliations:** Department of Aerospace Engineering & Engineering Mechanics, University of Cincinnati, Cincinnati, OH 45221, USA; chhabrad@mail.uc.edu (A.C.); venepajo@mail.uc.edu (J.R.V.)

**Keywords:** extended Kalman filter, adaptive filtering, disturbed environment, measurement noise covariance, nonlinear estimation

## Abstract

An accurate and reliable positioning system (PS) is a significant topic of research due to its broad range of aerospace applications, such as the localization of autonomous agents in GPS-denied and indoor environments. The PS discussed in this work uses ultra-wide band (UWB) sensors to provide distance measurements. UWB sensors are based on radio frequency technology and offer low power consumption, wide bandwidth, and precise ranging in the presence of nominal environmental noise. However, in practical situations, UWB sensors experience varying measurement noise due to unexpected obstacles in the environment. The localization accuracy is highly dependent on the filtering of such noise, and the extended Kalman filter (EKF) is one of the widely used techniques. In varying noise situations, where the obstacles generate larger measurement noise than nominal levels, EKF cannot offer precise results. Therefore, this work proposes two approaches based on EKF: sequential adaptive EKF and piecewise adaptive EKF. Simulation studies are conducted in static, linear, and nonlinear scenarios, and it is observed that higher accuracy is achieved by applying the proposed approaches as compared to the traditional EKF method.

## 1. Introduction

An accurate and reliable positioning system (PS) is currently a significant topic of research due to its broad range of applications, such as robotics, supermarkets, and accessibility aids for the visually impaired. The positioning of objects depends on localization techniques for active navigation. It can be defined as the concept of locating objects using devices like cameras, ultrasonic sensors, or ultra-wide band (UWB) ranging sensors. This study considers UWB sensors for the development of a robust PS. Generally, localization performance is affected by measurement noise caused by environmental factors. This can be improved with estimation techniques, and the Kalman filter (KF) is the most common one. In the presence of obstacles, however, the measurement noise may increase beyond the nominal environmental noise characteristics. The traditional approaches are typically unable to estimate the system states accurately in such situations. In the presence of such interference, the accuracy of localization and its robustness are the primary benchmarks of concern for the performance improvement of a PS.

A number of studies have been conducted to improve the performance of estimation techniques for localization and navigation in noisy environments. Choliz et al. [1] compared the various localization methods, including least squares (LS) with distance contraction, weighted LS with multi-dimensional scaling, extended KF (EKF), and particle filter (PF). Banerjee [2] presented a scheme to improve the precision of UWB sensor measurements by applying a PF to a noise model that attempts to identify the noise for a local PS. Assa and Plataniotis [3] proposed an adaptive KF to approximate the measurement and process noise covariance distributions through finite sampling under the assumption that it is not known a priori. Nguyen and Guillemin [4] presented a similar approach using multiple KFs, and an LS is applied to estimate the process noise covariance. Akhlaghi et al. [5] applied the adaptive filtering approach to estimate measurement and process noise covariance matrices using the covariance matching technique. These studies concerning noise reduction lack the consideration of random interference and the adaptive nature of the measurement noise covariance matrix to account for the aforementioned random interference. Fu et al. [6] proposed the application of three different estimation techniques based on the federated KF structure. In this method, the measurement-based adaptive KF algorithm and the improved Suge–Husa algorithm were proposed within the federated structure. Although this method provides improved performance, the modeling of three different algorithms for the purposes of localization in the field of robotics is undesirable due to the high computational burden. Furthermore, this approach mainly focuses on the application to ships in the absence of the satellite navigation system. Eroglu et al. [7] proposed the updating of the measurement noise covariance matrix based on a scaling factor. However, this approach mainly focused on the application to visible light communication-based localization, where the number of anchors is constantly changing, and the method adapts the measurement noise based on these changes. A fuzzy-based updating method was proposed by Woo for the measurement noise covariance matrix based on a number of inputs including the innovation vector of EKF, the position dilution of precision, and the number of receivable satellites [8]. In fuzzy-based systems containing many inputs; however, there are several rules and membership functions that need to be optimized to obtain a generalized fuzzy system that offers consistent performance. Most of the above methods focus on the estimation of the process and measurement noise covariance matrices leading to a standard formulation for most of the adaptive estimation techniques. Furthermore, to present substantial evidence as to why this standard formulation is unsuitable for the estimation of noise covariance matrices, Dunik et al. [9] provided a summary of the various methods for parameter estimation and prediction error-based correlation. Geng et al. [10] proposed the adaptive cubature KF (CKF) for attitude and heading estimation applied to indoor PSs based on the smart phone-embedded MARG sensors. The core concept of the proposed approach is to apply fading and limited memory-weighted methods to CKF. Although the proposed method provides improved performance over the existing methods, the application of adaptive weights to estimate the noise covariance matrices over the most recent measurements increases the computational burden of the approach. Huang et al. [11] proposed the variational Bayesian method-based adaptive KF for cooperative localization in scenarios with inaccurate prior information, where the expectation–maximization algorithm is used to estimate the prediction error covariance matrix. An alternate iteration strategy was also defined to further reduce the computational complexity of the proposed method.

This study does not primarily focus on the estimation of the noise covariance matrices, instead it proposes an approach that can provide reliable estimates of the target object location in a sensor-disturbed environment by updating the noise covariance. Regardless of the inaccurate measurement due to the disturbances, uncertainty in the estimation of states is reduced by updating the measurement noise covariance based on previous measurements. Two methods are presented. The first one is the *piecewise-adaptive EKF* (PA-EKF) technique. In this method, EKF adapts to the noise by updating the measurement noise covariance matrix after a batch of measurements has been collected. The second approach, the *sequentially-adaptive EKF* (SA-EKF) technique, shifts the batch by adding a new measurement and removing the oldest one rather than waiting for a completely new batch of measurements. In this way, the methods can adapt to the constantly changing environments and offer a more robust estimation technique for all kinds of PSs. Another primary advantage of these methods is that they are easily applicable to both KF and EKF techniques. This allows such modification to be generalized for several different situations. To validate the performance of the proposed approaches, multiple simulation studies are conducted in different scenarios, such as static, linear, and nonlinear motions. Furthermore, some analysis has been researched on selecting the batch sizes for further improvement in performance.

The paper is organized as follows: Section 2 provides a background on the preliminaries for this paper. Section 3 introduces the proposed methodologies. Simulation studies for validating the proposed approaches are defined in Section 4, and the corresponding results are discussed in Section 5. Finally, the conclusions are drawn in Section 6.

## 2. Preliminaries

This section addresses the sensor and types of disturbances considered for this study and the EKF structure.

### 2.1. UWB Sensors

With many major technology companies, such as Apple, Samsung, Decawave (Qorvo), and Xiaomi investing heavily in the UWB technology, it has become a popular option among the many ranging sensors available today. Indeed, UWB sensors outperform the alternatives, like Bluetooth and Wi-Fi, due to their distinctive characteristics, such as low cost, low power consumption, multi-path resistance, high data rate, and relatively accurate precision [12]. The UWB sensor modeled in this study is based on the Decawave DWM1001 module. This sensor provides distance measurements between each tag and nearby anchors. The sensors have a line-of-sight range of approximately 60 m with a nominal accuracy of around 10 cm. The operating frequency range for these sensors is 3.5–6.5 GHz with a data rate of up to 6.8 Mb/s. Although UWB sensors work well without any modification, they are highly susceptible to measurement noise arising out of environmental factors, such as obstacles [13] and weather [14]. In addition to the nominal noise, UWB sensors experience much larger measurement errors when interfered with metallic structures, other threatening objects, and other wireless systems [15,16]. Since these unanticipated errors compromise the accuracy of measurement, the PS with UWB sensors lacks credibility in such situations.

For the PS discussed in this study, the nodes are classified into tags and anchors. The anchor is a stationary UWB sensor that has a manually set location in three-dimensional (3D) space. On the other hand, the tag is the sensor attached to the object being tracked. To estimate the position of a tag on the ground, for example, at least three anchors are needed [17]. Therefore, in this study, three UWB sensors are used as anchors, and one UWB sensor is used as a tag.

### 2.2. Disturbance Model

Normally, the nominal sensor noise $\nu_{k}$ is generally assumed to follow a Gaussian distribution that has a zero mean. The noise is then defined based on some predefined covariance (*R*) of the sensor measurement as given below [18]:

(1) $$\begin{array}{c} \nu_{k}∼N\left(0,R\right). \end{array}$$

**Figure 1:**
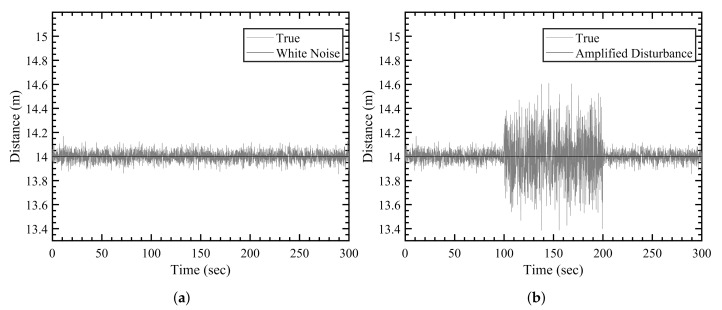
Disturbed measurements. (**a**) White noise, (**b**) amplified disturbance.

Therefore, for given distance measurements, the sensor generally gives bounded disturbed measurements in the presence of nominal sensor noise as shown in Figure 1a. Hereon, white noise will be used to refer to the aforementioned zero-mean white Gaussian noise. Since the various environmental obstacles, such as metallic objects, can increase the noise amplitude beyond the nominal specifications, the amplified disturbance affecting the sensor measurement data is written as follows:

(2) $$\begin{array}{c} \nu_{k}∼\eta \times N\left(0,R\right), \end{array}$$

where η is the noise amplification factor. The disturbed measurement is then shown in Figure 1b, and its comparison with the white noise shows the amplified errors in the distance measurements.

### 2.3. Extended Kalman Filter

EKF is one of the most popular estimation techniques applied to localization and navigation problems for the reduction of sensor measurement noise. It is a variation of the traditional KF algorithm for nonlinear systems. It estimates a system’s state by predicting *a priori* estimates and then updating these predictions by taking into consideration the new measurements to obtain *a posteriori* estimates. The model considers the disturbance which is generally modeled as zero-mean Gaussian white noise. The modeling equations for EKF are given as [19]

(3) $$x_{k+1}=f\left(x_{k},u_{k}\right)\;+\;w_{k},\;\;w_{k}∼N\left(0,Q\right),$$

(4) $${\tilde{y}}_{k}=h\left(x_{k}\right)\;+\;\nu_{k},\;\;\;\nu_{k}∼N\left(0,R\right),$$

where $f\left(x_{k},u_{k}\right)$ and $h\left(x_{k}\right)$ are the nonlinear functions that make up the system model definition, and $w_{k}$ and $\nu_{k}$ are the zero-mean white Gaussian noise vectors corresponding to the process noise and the measurement noise. The EKF algorithm then operates in three stages [19]. In the initialization stage, $\hat{x}\left(t_{0}\right)$ indicates the initial state estimate, and $P_{0}$ is the initial error covariance matrix. Then, the estimation stage determines the *a posteriori* estimates ${\hat{x}}_{k}^{+}$ and $P_{k}^{+}$ using the *a priori* estimates ${\hat{x}}_{k}^{-}$ and $P_{k}^{-}$. In order to do this, first the Kalman gain $K_{k}$ is determined at every time step. Based on the *a posteriori* estimates, the *a priori* estimates for the next time step ${\hat{x}}_{k+1}^{-}$ and $P_{k+1}^{-}$ are then determined in the propagation stage.

## 3. Proposed Methodologies

**Figure 2:**
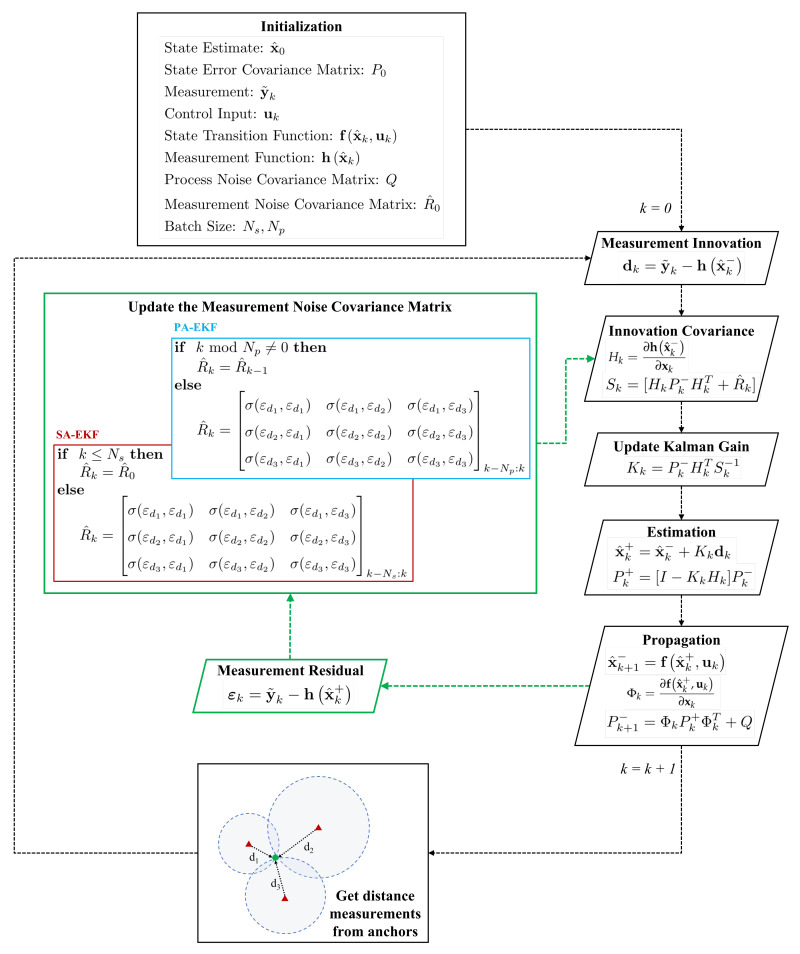
Proposed adaptive EKF approaches.

EKF assumes that the measurement noise covariance is constant over time. In theory, this assumption could be applicable under circumstances where the measurement noise remains nominal which is generally provided as a sensor specification obtained via statistical data analysis. Since this analysis provides a constant value known as the standard deviation of the sensor’s noise, the measurement noise covariance has a constant value. However, the measurements obtained from sensors can exhibit random interference due to the presence of obstacles like metallic structures, other wireless systems, etc., causing the standard deviation of the noise distribution to be different than what is stated. Thus, the conventional techniques, where the measurement noise covariance is kept constant, become unreliable. The proposed methods update the measurement noise covariance matrix over time in two different ways. Figure 2 illustrates the basic principle of the proposed methodologies. As evident from the figure, it must be noted that the update process is independent of the choice of KF or EKF. In the following sections, one describes how the measurement noise covariance is updated over time in both the proposed methodologies. It is important to highlight that the proposed methods are not intended towards estimation of the covariance matrix, instead focused on updating the covariance matrix to obtain better state estimates.

### 3.1. Piecewise-Adaptive Extended Kalman Filter

PA-EKF allows EKF to adapt to the noise by updating the measurement noise covariance matrix after a certain number of measurements has been collected. These measurements are referred to as a batch, and the number of measurements in a batch is indicated by $N_{p}$. This is known as a piece-wise batch, since the batch is updated after a fixed number of measurements has been obtained as shown in Figure 2. Depending on the situation, $N_{p}$ can be tuned to increase the performance of PA-EKF. Since the measurement noise covariance is intentionally updated over time, it is indicated by ${\hat{R}}_{k}$ instead of *R* to clearly distinguish between the conventional and adaptive methods.

The initial measurement error covariance matrix is expressed as follows:

(5) $$\begin{array}{c} {\hat{R}}_{0}=I\sigma_{m}^{2}, \end{array}$$

where $I\in R_{n\times n}$ is an identity matrix with *n* equal to the number of measurements, and $\sigma_{m}$ is the standard deviation of nominal measurement noise. It is important to note that PA-EKF does not provide an estimate corresponding to every measurement. After every $N_{p}$ measurement, the measurement error covariance is calculated as

(6) $$\begin{array}{c} {\hat{R}}_{k}=\left\{\begin{array}{cc} {\left[\begin{array}{ccc} \sigma (\varepsilon_{d_{1}},\varepsilon_{d_{1}}) & \sigma (\varepsilon_{d_{1}},\varepsilon_{d_{2}}) & \sigma (\varepsilon_{d_{1}},\varepsilon_{d_{3}})\\ \sigma (\varepsilon_{d_{2}},\varepsilon_{d_{1}}) & \sigma (\varepsilon_{d_{2}},\varepsilon_{d_{2}}) & \sigma (\varepsilon_{d_{2}},\varepsilon_{d_{3}})\\ \sigma (\varepsilon_{d_{3}},\varepsilon_{d_{1}}) & \sigma (\varepsilon_{d_{3}},\varepsilon_{d_{2}}) & \sigma (\varepsilon_{d_{3}},\varepsilon_{d_{3}}) \end{array}\right]}_{k-N_{p}:k},\; & \mathrm{if}\;k\;\mathrm{mod}\;N_{p}=0,\\ {\hat{R}}_{k-1},\; & \mathrm{if}\;k\;\mathrm{mod}\;N_{p}\neq 0, \end{array}\right. \end{array}$$

where $\varepsilon ={\left[\varepsilon_{d_{1}}\;\;\varepsilon_{d_{2}}\;\;\varepsilon_{d_{3}}\right]}^{T}$ denotes the measurement residual of the three distance measurements, and ${\hat{y}}_{k}=h\left({\hat{x}}_{k}^{+}\right)$. Moreover, the expression for covariance is given as follows:

(7) $$\begin{array}{c} \sigma \left(a,b\right)=\frac{1}{n-1}\sum_{i=1}^{n}\left(a_{i}-\bar{a}\right)\left(b_{i}-\bar{b}\right), \end{array}$$

where *a* and *b* are two random variables containing *n* number of data values, and $\bar{a}$ and $\bar{b}$ represent their mean. The remaining expressions of EKF remain unchanged. After the measurement noise covariance is updated, the Kalman gain can be calculated similar to EKF by replacing *R* with ${\hat{R}}_{k}$.

PA-EKF method works best when the measurement update rate is high. When the new measurements come at slow time intervals, waiting for the new batch can cause the measurement noise covariance to worsen and have a negative impact on the state estimation. This is the reason why PA-EKF is preferred for sensors where the measurements are obtained at small time intervals. For example, if the measurement rate is 0.01 Hz, then with a batch size of 50, it updates the measurement noise covariance after every 0.5 s. On the other hand, if the update rate of a given sensor is 1 Hz, it has to wait for every 50 s to update PA-EKF making it unsuitable for real-time application for that sensor.

### 3.2. Sequentially-Adaptive Extended Kalman Filter

When compared to PA-EKF, SA-EKF method resolves the update problem by modifying the algorithm to shift the batch sequentially rather than obtain a new batch for every $N_{p}$ measurements. As it can be seen in Figure 2, the shifting batch size is indicated by $N_{s}$ to avoid confusion. This approach operates by dropping the oldest measurements and adding the latest one, always keeping constant $N_{s}$ measurements in the sequential batch. The initial measurement error covariance matrix is expressed similarly as given in Equation (5). In SA-EKF, after obtaining $N_{s}$ measurements, ${\hat{R}}_{k}$ is updated corresponding to every new measurement and is defined as

(8) $$\begin{array}{c} {\hat{R}}_{k}=\left\{\begin{array}{cc} {\hat{R}}_{0},\; & \mathrm{if}\;k<N_{s},\\ {\left[\begin{array}{ccc} \sigma (\varepsilon_{d_{1}},\varepsilon_{d_{1}}) & \sigma (\varepsilon_{d_{1}},\varepsilon_{d_{2}}) & \sigma (\varepsilon_{d_{1}},\varepsilon_{d_{3}})\\ \sigma (\varepsilon_{d_{2}},\varepsilon_{d_{1}}) & \sigma (\varepsilon_{d_{2}},\varepsilon_{d_{2}}) & \sigma (\varepsilon_{d_{2}},\varepsilon_{d_{3}})\\ \sigma (\varepsilon_{d_{3}},\varepsilon_{d_{1}}) & \sigma (\varepsilon_{d_{3}},\varepsilon_{d_{2}}) & \sigma (\varepsilon_{d_{3}},\varepsilon_{d_{3}}) \end{array}\right]}_{k-N_{s}:k},\; & \mathrm{if}\;k\geq N_{s}. \end{array}\right. \end{array}$$

Similar to PA-EKF, the Kalman gain is calculated as before by replacing *R* with ${\hat{R}}_{k}$. Note that both PA-EKF and SA-EKF highlight the straightforward nature of the approaches in being able to modify the conventional EKF approach by updating the measurement noise covariance matrix.

SA-EKF is advantageous compared to PA-EKF, in the sense that it provides better performance independent of the measurement update rate. SA-EKF works by taking the last $N_{s}$ measurements at every time step to update the measurement noise covariance matrix after obtaining the first batch. Contrary to PA-EKF, when the measurement rate is high, SA-EKF may lead to a higher computational burden and does not necessarily need to update the noise covariance at high update rates. For example, if the update rate of the sensor is 100 Hz, then the updating process will happen every 0.01 s after the first 0.5 s. This may be unnecessary for several applications. However, SA-EKF still has the advantage of being operational and efficient in all kinds of scenarios independent of the sensor update rate.

## 4. Simulation Study

**Figure 3:**
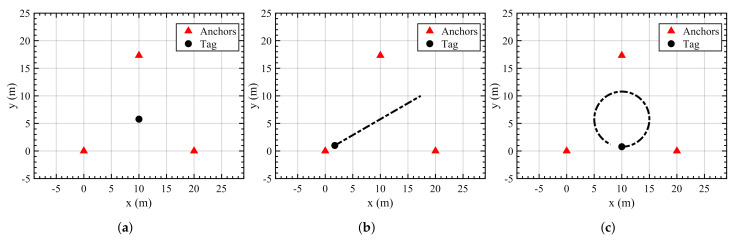
Simulation environments. (**a**) Static tag, (**b**) tag in linear motion, (**c**) tag in nonlinear motion.

Figure 3 represents a 2D environment modeled for this work consisting of three anchors and one tag. The three anchors are kept in an equilateral triangular formation to observe similar trends when the anchors are subjected to amplified disturbances. Figure 3 contains three representations for each of the simulation cases discussed in Section 4.1. The sensor characteristics like the measurement update rate, process noise, and nominal measurement noise are chosen to model the Decawave UWB sensor [20]. For the amplified disturbances affecting the measurement signal, the noise amplification factor (η) is chosen as 4, so as to observe noticeable trends between the traditional and the proposed adaptive EKF approaches [21]. See Appendix A for further analysis regarding the performance of the proposed approaches with different values of η.

Furthermore, the batch size for the proposed approaches should be selected considering several factors as follows:

- One of the important factors is the *measurement update rate*. If the measurement update rate is low, then a large batch is required for SA-EKF. However, if a large batch size is selected, the performance of PA-EKF is reduced as updating process of the algorithm will be delayed.
- The *acceptable delay* before the first update of the measurement noise covariance matrix also impacts the choice of the batch size. When the batch size is too big, the time taken for the first update can increase, especially if the measurement update rate is not high.
- Another important factor is the *available system memory*. Usually these sensors are connected to micro controller units (MCUs), which have limited system memory [22]. Therefore, the large batch size is dependent on the available memory to store and process the measurement data.

Generally, the type of problem helps the user determine the correct batch size. In the current problem, performance is impacted by the above factors as the bigger batch size can delay the first update rate and a small batch size does not preserve the historical measurement information. Furthermore, the limited system memory does not allow for bigger batch sizes in practical situations. Therefore, through the aforementioned analysis, the values for $N_{s}$ and $N_{p}$ are chosen as 50 to compensate for the acceptable delay, the update rate, and the available system memory in most MCUs [22]. The various parameters that define the sensor characteristics considered are summarized in Table 1.

### 4.1. Simulation Cases

Three simulation cases are considered: static, linear, and nonlinear. In all cases, the anchor positions are kept constant, and the nominal sensor noise characteristics remain the same. Furthermore, the sensors are modeled to experience certain disturbances during the operation, which leads the measurement noise to be amplified by a certain η. It should be noted that, in the following cases, the initial value for the state error covariance matrix ($P_{0}$) is arbitrarily chosen to depict some initial confidence in the estimation model.

#### 4.1.1. Case 1: Static Tag

In the static case, the tag remains at a constant position ${\left[x_{k}\;\;y_{k}\right]}^{T}$; and therefore, the control input vector applied to the system is expressed as $u_{k}={\left[0\;\;0\right]}^{T}$. Due to the nature of the static tag (See Figure 3a), the components of EKF are written as follows:

(9) $$\begin{array}{ccc} f\left(x_{k},u_{k}\right) & = & {\left[\begin{array}{cc} x_{k} & y_{k} \end{array}\right]}^{T}, \end{array}$$

(10) $$\begin{array}{ccc} h\left(x_{k}\right) & = & \left[\begin{array}{c} d_{1_{k}}\\ d_{2_{k}}\\ d_{3_{k}} \end{array}\right]=\left[\begin{array}{c} {\left[{\left(x_{1}-x_{k}\right)}^{2}+{\left(y_{1}-y_{k}\right)}^{2}\right]}^{1/2}\\ {\left[{\left(x_{2}-x_{k}\right)}^{2}+{\left(y_{2}-y_{k}\right)}^{2}\right]}^{1/2}\\ {\left[{\left(x_{3}-x_{k}\right)}^{2}+{\left(y_{3}-y_{k}\right)}^{2}\right]}^{1/2} \end{array}\right]. \end{array}$$

Here, the given expressions for the aforementioned system lead to the following state transition and observation matrices:

(11) $$\begin{array}{ccc} \Phi_{k} & = & \left[\begin{array}{cc} 1 & 0\\ 0 & 1 \end{array}\right], \end{array}$$

(12) $$\begin{array}{ccc} H_{k} & = & \left[\begin{array}{cc} \frac{x_{k}-x_{1}}{d_{1_{k}}} & \frac{y_{k}-y_{1}}{d_{1_{k}}}\\ \frac{x_{k}-x_{2}}{d_{2_{k}}} & \frac{y_{k}-y_{2}}{d_{2_{k}}}\\ \frac{x_{k}-x_{3}}{d_{3_{k}}} & \frac{y_{k}-y_{3}}{d_{3_{k}}} \end{array}\right]. \end{array}$$

The system is then represented as given in Equations (3) and (4), where the measurements are the noisy distance values corresponding to each anchor with the following initial conditions:

$$\begin{array}{ccc} {\hat{x}}_{0} & = & {\left[\begin{array}{cc} 10\;m & 10\;m \end{array}\right]}^{T},\\ P_{0} & = & \mathrm{diag}(\left[\begin{array}{cc} 10^{-4} & 10^{-4} \end{array}\right]),\\ {\hat{R}}_{0} & = & \sigma {{}_{m}}^{2}I_{3\times 3},\\ Q & = & \sigma_{p}^{2}I_{2\times 2}. \end{array}$$

#### 4.1.2. Case 2: Linear Motion

In the linear motion case, the tag moves in a straight line with a constant velocity ${\left[v_{x_{k}}\;\;v_{y_{k}}\right]}^{T}$ (See Figure 3b). Similar to the static case, there is no control in the linear case. The relationship between position and velocity is linear due to constant velocity; and therefore, the state vector is chosen as:

(13) $$\begin{array}{c} x_{k}={\left[\begin{array}{cccc} x_{k} & y_{k} & v_{x_{k}} & v_{y_{k}} \end{array}\right]}^{T}. \end{array}$$

Identical to the previous case, the distance values obtained using the UWB sensors are used as the measurement vector; and therefore, the system model components are written as

(14) $$\begin{array}{ccc} f\left(x_{k},u_{k}\right) & = & \left[\begin{array}{c} x_{k}+v_{x_{k}}\Delta t\\ y_{k}+v_{y_{k}}\Delta t\\ v_{x_{k}}\\ v_{y_{k}} \end{array}\right], \end{array}$$

where $\Delta t=t_{k}-t_{k-1}$ is the time step. The corresponding state transition and observation matrices are found as

(15) $$\begin{array}{ccc} \Phi_{k} & = & \left[\begin{array}{cccc} 1 & 0 & \Delta t & 0\\ 0 & 1 & 0 & \Delta t\\ 0 & 0 & 1 & 0\\ 0 & 0 & 0 & 1 \end{array}\right], \end{array}$$

(16) $$\begin{array}{ccc} H_{k} & = & \left[\begin{array}{cccc} \frac{x_{k}-x_{1}}{d_{1_{k}}} & \frac{y_{k}-y_{1}}{d_{1_{k}}} & 0 & 0\\ \frac{x_{k}-x_{2}}{d_{2_{k}}} & \frac{y_{k}-y_{2}}{d_{2_{k}}} & 0 & 0\\ \frac{x_{k}-x_{3}}{d_{3_{k}}} & \frac{y_{k}-y_{3}}{d_{3_{k}}} & 0 & 0 \end{array}\right]. \end{array}$$

The system is then represented as given in Equations (3) and (4) with the following initial conditions:

$$\begin{array}{ccc} {\hat{x}}_{0} & = & {\left[\begin{array}{cccc} 1\;m & 1\;m & 0.1\;m/s & 0.1\;m/s \end{array}\right]}^{T},\\ P_{0} & = & \mathrm{diag}(\left[\begin{array}{cccc} 10^{-4} & 10^{-4} & 10^{-5} & 10^{-5} \end{array}\right]),\\ Q & = & \sigma_{p}^{2}I_{4\times 4}. \end{array}$$

#### 4.1.3. Case 3: Nonlinear Motion

In this scenario, the tag moves in a circular path, which is a nonlinear trajectory; and therefore, the heading angle (θ) is introduced as a new state component (See Figure 3c). The state vector is then written as

(17) $$\begin{array}{c} x_{k}={\left[\begin{array}{ccc} x_{k} & y_{k} & \theta_{k} \end{array}\right]}^{T}. \end{array}$$

The measurements again contain the distance information obtained from the UWB sensors. The control input vector applied to the system is expressed as

(18) $$\begin{array}{c} u_{k}={\left[\begin{array}{cc} v & \omega \end{array}\right]}^{T}, \end{array}$$

where *v* is the constant translational velocity, and ω is the constant angular velocity applied to the model. Based on this, the system model is represented as given in Equations (3) and (4) with the following nonlinear components:

(19) $$\begin{array}{ccc} f\left(x_{k},u_{k}\right) & = & \left[\begin{array}{c} x_{k}+v\;\Delta t\;\cos\left(\theta_{k}\right)\\ y_{k}+v\;\Delta t\;\sin\left(\theta_{k}\right)\\ \theta_{k}+\omega \;\Delta t \end{array}\right]. \end{array}$$

These are then used to determine state transition and observation matrices:

(20) $$\begin{array}{ccc} \Phi_{k} & = & \left[\begin{array}{ccc} 1 & 0 & -v\Delta t\sin\theta_{k}\\ 0 & 1 & v\Delta t\cos\theta_{k}\\ 0 & 0 & 1 \end{array}\right], \end{array}$$

(21) $$\begin{array}{ccc} H_{k} & = & \left[\begin{array}{ccc} \frac{x_{t}-x_{1}}{d_{1_{k}}} & \frac{y_{t}-y_{1}}{d_{1_{k}}} & 0\\ \frac{x_{t}-x_{2}}{d_{2_{k}}} & \frac{y_{t}-y_{2}}{d_{2_{k}}} & 0\\ \frac{x_{t}-x_{3}}{d_{3_{k}}} & \frac{y_{t}-y_{3}}{d_{3_{k}}} & 0 \end{array}\right], \end{array}$$

with the following initial conditions:

$$\begin{array}{ccc} {\hat{x}}_{0} & = & {\left[\begin{array}{ccc} 10\;m & 5\;m & 0\;\mathrm{rad} \end{array}\right]}^{T},\\ u_{0} & = & {\left[\begin{array}{cc} 0.1571\;m/s & 0.0314\;\mathrm{rad}/s \end{array}\right]}^{T},\\ P_{0} & = & \mathrm{diag}(\left[\begin{array}{ccc} 10^{-4} & 10^{-4} & 10^{-3} \end{array}\right]),\\ Q & = & \sigma_{p}^{2}I_{3\times 3}. \end{array}$$

### 4.2. Disturbance Scenarios

In addition to the aforementioned simulation cases, three disturbance scenarios are considered to compare the results between the proposed methods and the traditional EKF technique. Here, the disturbances are not specified, instead, the study focuses on the disturbed measurements. The amplitude of the disturbed measurement signal (η) is defined by the amount of noise generated by the environmental obstacles. See Appendix A for further analysis. Note that these disturbances refer to the disturbed measurement signals; and therefore, they have the same frequency as the measurement signal (10 Hz). Furthermore, the width of the disturbance is determined by environmental factors like obstacles and how long do they disturb the sensor which can be random. The simulation study is conducted by applying the proposed methodologies to all simulation cases with anchors subjected to the following types of disturbances.

#### 4.2.1. Nominal Disturbance

**Figure 4:**
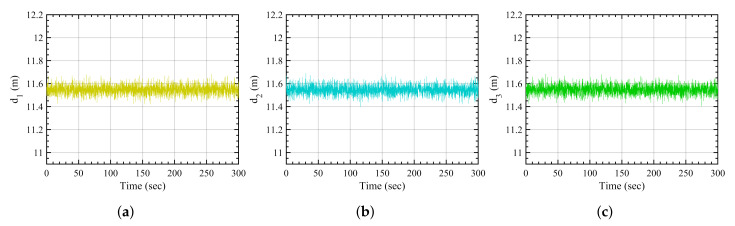
Nominal disturbance-based measurements. (**a**) Anchor 1, (**b**) anchor 2, (**c**) anchor 3.

The nominal disturbance scenario is defined as anchors being subjected to the white noise without any amplified disturbances caused by obstacles in the environment. Figure 4 shows the white noise-affected distance measurements from the three anchors.

#### 4.2.2. Isolated Disturbance

**Figure 5:**
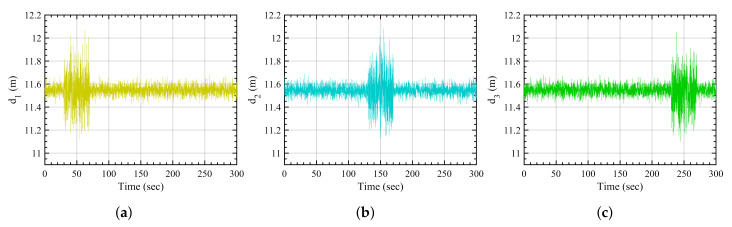
Isolated disturbance-based measurements. (**a**) Anchor 1, (**b**) anchor 2, (**c**) anchor 3.

In this scenario, all of the anchors are subjected to isolated amplified disturbances meaning that at a given point of time, only one anchor is subjected to such disturbance as shown in Figure 5. Starting from anchor 1, the disturbance is then removed from anchor 1 and shifted to anchor 2, and so on. Each sensor is disturbed for 40 s at different times. This can be a representation of a single obstacle moving through the environment.

#### 4.2.3. Simultaneous Disturbance

**Figure 6:**
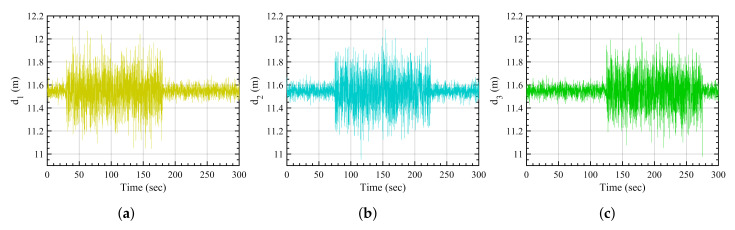
Simultaneous disturbance-based measurements. (**a**) Anchor 1, (**b**) anchor 2, (**c**) anchor 3.

The final scenario is the simultaneous disturbance scenario, where all anchors are subjected to the amplified disturbance in a sequential manner. First, anchor 1 is disturbed followed by anchor 2 while anchor 1 is being subjected to disturbance. Then, anchor 3 is disturbed while anchors 1 and 2 are still being disturbed. This is followed by disturbance being removed from these anchors one by one. The duration of the disturbed measurements is 150 s each starting at 30 s, 75 s, and 125 s for anchors 1, 2, and 3, respectively. This scenario is selected to observe a definitive trend caused by an environment affected by a large amount of disturbance. The disturbance-affected distance measurements from the three anchors are shown in Figure 6.

## 5. Results and Discussion

For the three simulation cases described along with the three disturbance scenarios, a total of nine settings are simulated. The results of the traditional and the proposed methods are compared to highlight the improvement offered by PA-EKF and SA-EKF as opposed to the traditional EKF.

### 5.1. Static Case

**Figure 7:**
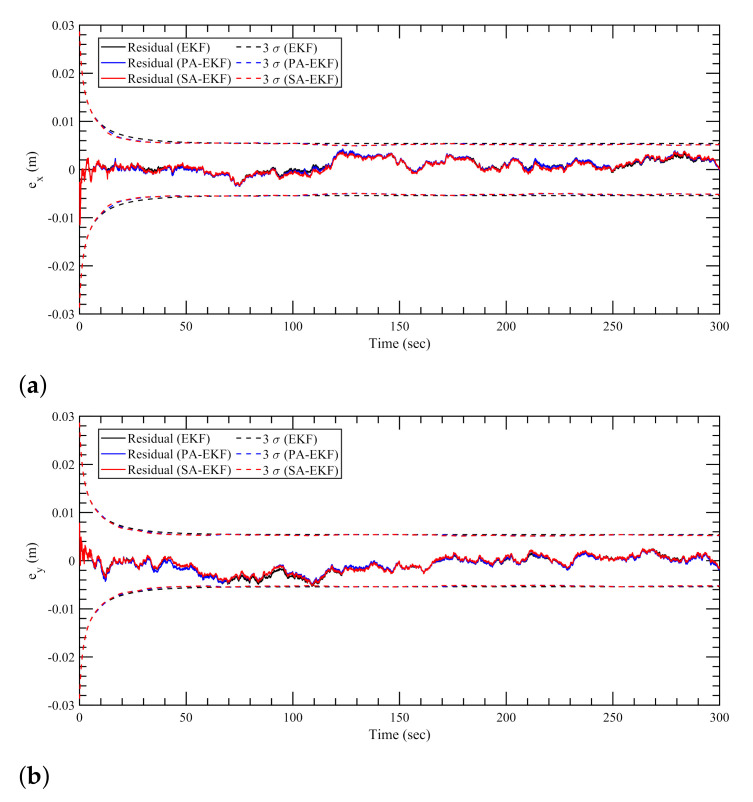
Estimation results for static tag with anchors subjected to nominal disturbances. (**a**) Error in *x*–direction, $e_{x}$, (**b**) error in *y*–direction, $e_{y}$.

**Figure 8:**
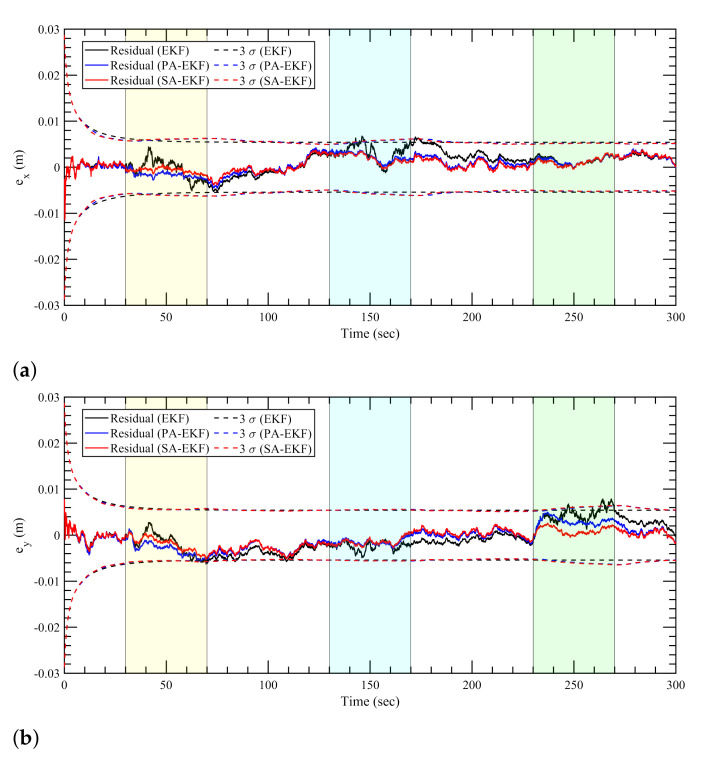
Estimation results for static tag with anchors subjected to isolated disturbances. (**a**) Error in *x*–direction, $e_{x}$, (**b**) error in *y*–direction, $e_{y}$.

**Figure 9:**
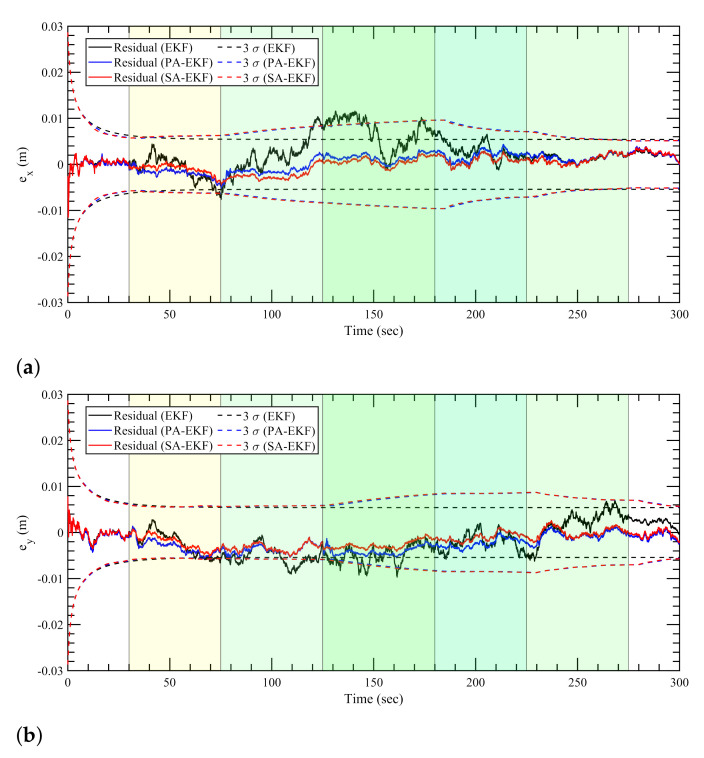
Estimation results for static tag with anchors subjected to simultaneous disturbances. (**a**) Error in *x*–direction, $e_{x}$, (**b**) error in *y*–direction, $e_{y}$.

Figure 7, Figure 8 and Figure 9 represent the estimation results for the static tag case with the anchors subjected to nominal, isolated, and simultaneous disturbances, respectively. It can be observed that, in Figure 7, during the period of nominal disturbance or white noise, EKF, PA-EKF, and SA-EKF perform equivalently, showing the similarities between the three methods. It is only under the effect of amplified disturbances that the proposed methods adapt to the situation and perform better than expected as represented by the EKF results. In Figure 8, it can be seen that the proposed methods have small residuals that lie within the 3σ boundaries whereas the EKF residuals go outside the 3σ boundaries. This is noticeable especially in Figure 8a between 140 and 180 s and in Figure 8b during the third disturbance between 230 and 270 s. Furthermore, under the influence of simultaneous disturbances, where all sensors are subjected to amplified disturbances at the same time, the proposed methods perform considerably better as shown in Figure 9. In Figure 9a, EKF cannot handle the situation where all three anchors are disturbed, and the errors stay outside the 3σ boundaries consistently. Even when the first and second anchors are disturbed together at around 75 s, the EKF residuals go outside the confidence region, whereas the residuals via the proposed methods stay well within the boundaries. In addition to the small residuals, it can be observed that the 3σ boundaries for PA-EKF and SA-EKF adjust based on the changes in the measurement noise. A trend can be observed where the 3σ boundaries first increase with the number of disturbed sensors, and then decreases as the disturbances are withdrawn. Even though the disturbances are applied in a static case where the tag does not move, EKF is unable to provide consistent results as compared to the proposed methods.

### 5.2. Linear Case

**Figure 10:**
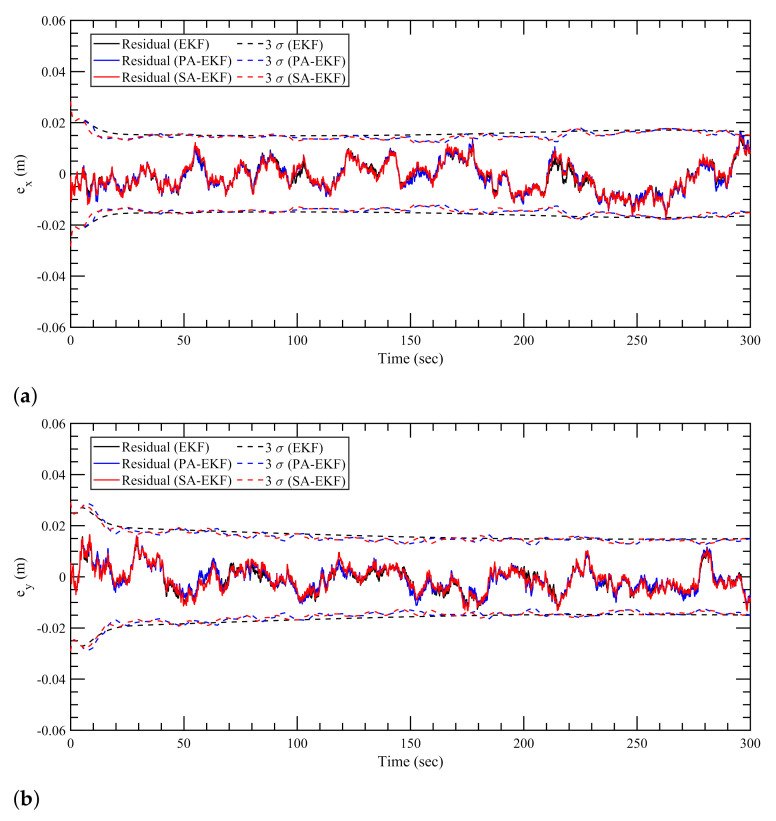
Estimation results for linear tag with anchors subjected to nominal disturbances. (**a**) Error in *x*–direction, $e_{x}$, (**b**) error in *y*–direction, $e_{y}$.

**Figure 11:**
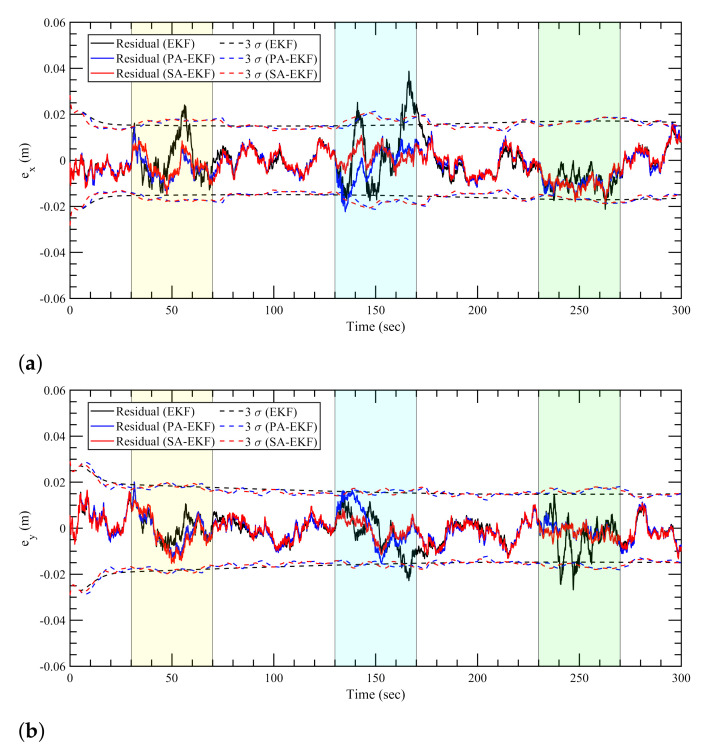
Estimation results for linear tag with anchors subjected to isolated disturbances. (**a**) Error in *x*–direction, $e_{x}$, (**b**) error in *y*–direction, $e_{y}$.

**Figure 12:**
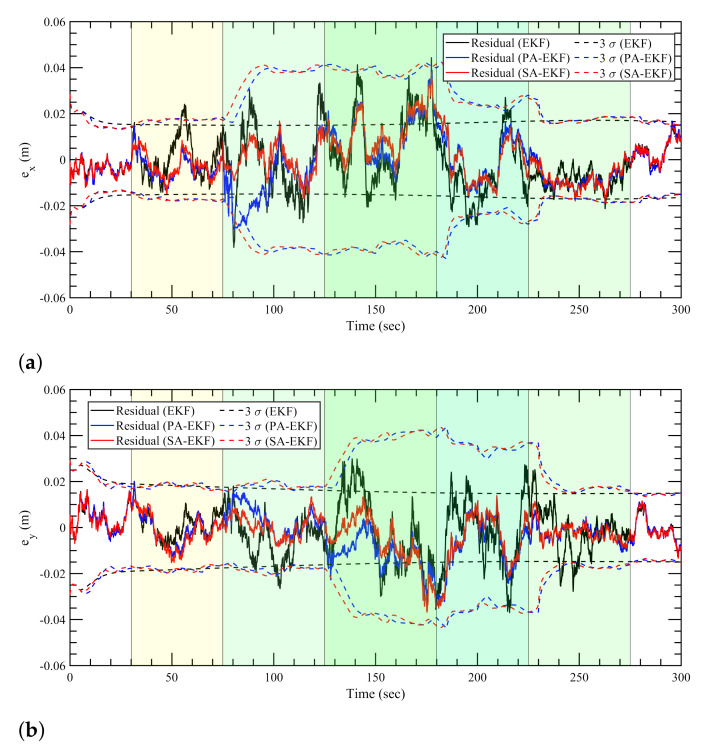
Estimation results for linear tag with anchors subjected to simultaneous disturbance. (**a**) Error in *x*–direction, $e_{x}$, (**b**) error in *y*–direction, $e_{y}$.

The estimation results for the linearly moving tag with the anchors subjected to nominal, isolated, and simultaneous disturbances are shown in Figure 10, Figure 11 and Figure 12, respectively. In the first observation, it can be seen that the results for the nominal case follow the same trend like the static tag case where all three approaches work similarly. It can be seen in Figure 11 and Figure 12 that during the period of amplified disturbances, the proposed methods adapt to the situation much better than EKF, similar to the static tag case. In Figure 11a, it can be seen that the error in *x*–position for the linearly moving tag, the proposed methods have errors within the 3σ boundaries. On the other hand, the errors within the EKF model are consistently high and go outside the 3σ boundaries especially when the amplified disturbance is applied to the three anchors at around 60 s, 140–170 s, and 260 s. This can also be seen in Figure 11b for the error in *y*–position during the second and third anchor disturbance scenarios at around 165 s and 240 s. Apart from the worse performance of EKF compared to the proposed methods, it can also be observed that PA-EKF residuals also go outside the confidence region sometimes, around 135 s in Figure 11a, and 32 s and 135 s in Figure 11b. This can largely be attributed to the slow update rate of PA-EKF compared to SA-EKF as the residuals of SA-EKF consistently stay within the 3σ boundaries. Moreover, since PA-EKF updates the measurement noise covariance matrix every 5 s, it starts adjusting to the amplified disturbance after some time, and then the residuals stay within the 3σ boundary.

When the anchors are subjected to amplified disturbances at the same time, the proposed methods perform considerably better as shown in Figure 12. In Figure 12a, when only the first anchor is disturbed at around 55 s, the EKF residuals go outside the 3σ boundaries. When the second anchor is disturbed at 75 s, both EKF and PA-EKF fail to adjust to the sudden changes, and therefore the residuals are bigger. Once adjusted, PA-EKF displays more robustness to the disturbance-caused measurement noise. On the other hand, SA-EKF consistently outperforms both methods as it stays within the 3σ boundaries. A similar trend can be observed for the *y*–position error, Figure 12b, as PA-EKF initially takes some time to adjust when all three anchors are disturbed. However, EKF cannot adjust properly; and therefore, larger errors are observed. Furthermore, the adaptive nature of the proposed methods can be seen in the simultaneous disturbance scenario as the 3σ boundaries first increase with the increasing disturbances in the environment, and then accordingly decrease as the disturbances are discontinued from individual anchors.

### 5.3. Nonlinear Case

**Figure 13:**
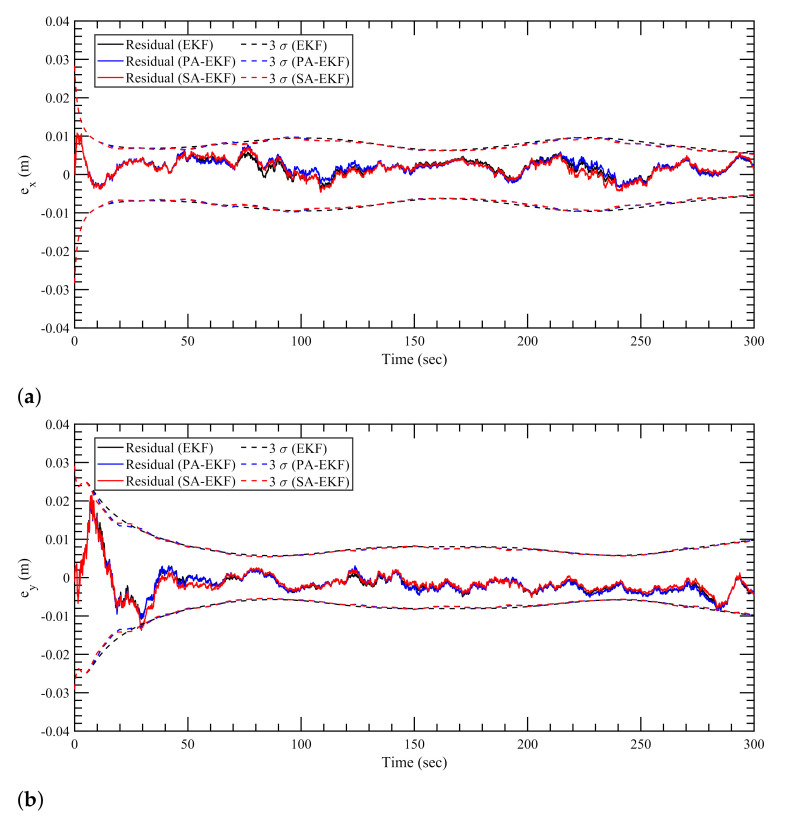
Estimation results for nonlinear tag with anchors subjected to nominal disturbances. (**a**) Error in *x*–direction, $e_{x}$, (**b**) error in *y*–direction, $e_{y}$.

**Figure 14:**
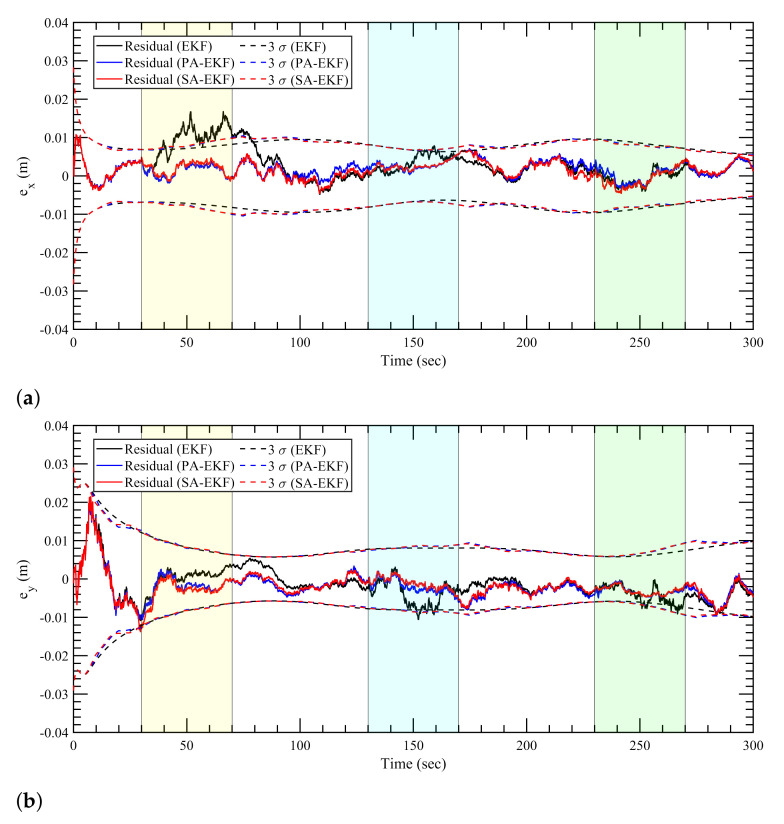
Estimation results for nonlinear tag with anchors subjected to isolated disturbances. (**a**) Error in *x*–direction, $e_{x}$, (**b**) error in *y*–direction, $e_{y}$.

For the nonlinear case, the three methods follow a consistent trend for the nominal case, similar to the static and linear cases as all three methods perform similar to each other in Figure 13. In Figure 14a, during the period of the first amplified disturbance between 40 s and 80 s, the proposed methods display robustness to the disturbance while the EKF residuals for the *x*–position go outside the 3σ boundaries. The amplified disturbance to the second anchor again causes the EKF residuals to go outside the 3σ boundaries at around 150 s. For the *y*–position errors, as shown in Figure 14b, the EKF residuals follow the same trend as the model is unable to adapt, and the residuals are large during the period of the second and third disturbances at around 150 and 250 s; and therefore, the residuals are not contained within the confidence interval.

**Figure 15:**
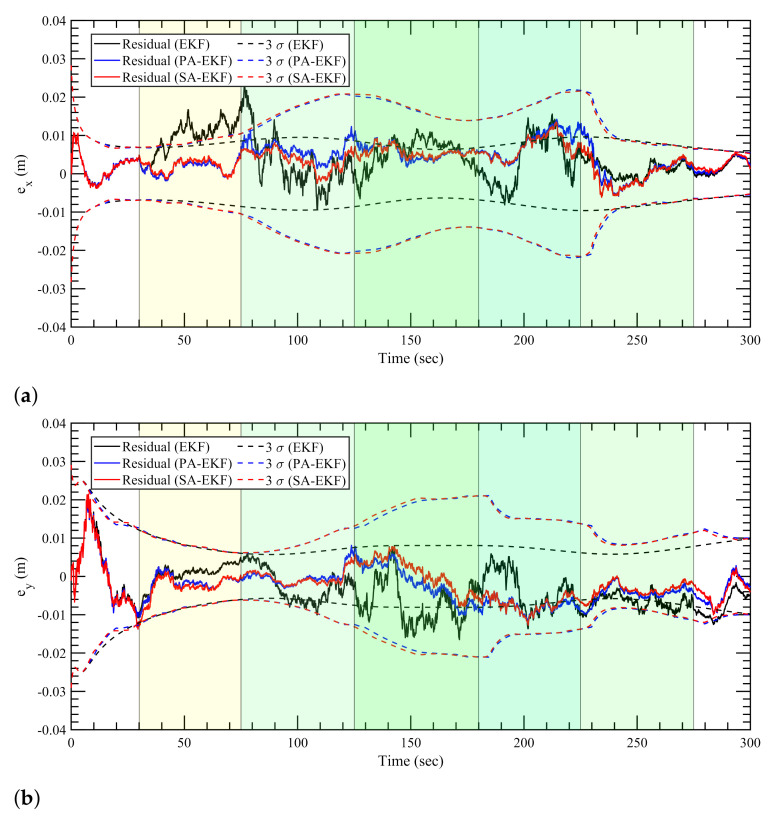
Estimation results for nonlinear tag subjected to simultaneous disturbances. (**a**) Error in *x*–direction, $e_{x}$, (**b**) error in *y*–direction, $e_{y}$.

In the simultaneous disturbance scenario, the advantages of the proposed methods are clearly evident again as the 3σ boundaries highlight the adaptive nature based on the changing disturbances in the environment. As soon as the disturbances are introduced to the first anchor, the EKF residuals magnify whereas SA-EKF and PA-EKF are able to keep the positioning errors close to zero. This is evident clearly in Figure 15a as the EKF residuals regularly go outside the confidence interval whereas the proposed methods show robustness to all stages of disturbances. In Figure 15b, once all three anchors are disturbed at 125 s, the EKF residuals consistently drop outside the 3σ boundaries, whereas PA-EKF and SA-EKF are able to attune to the new disturbances as the residuals stay small and within their respective 3σ boundaries.

It is interesting to notice, however, that the SA-EKF residuals also momentarily go outside the 3σ boundaries at around 30 s in Figure 14b and Figure 15b. Even though this behavior coincides with the start of disturbance to anchor 1, it can be attributed as an outlier situation as the same can also be observed in Figure 13b. Furthermore, there is no other evidence of SA-EKF being unable to adapt to the disturbance.

### 5.4. Monte Carlo Simulations

To validate the performance of the approaches proposed, the results of the static, linear, and nonlinear scenarios are simulated 1000 times in all disturbance cases. Note that the batch size for SA-EKF and PA-EKF ($N_{s}$ and $N_{p}$) and the noise amplification factor (η) are the same as given in Table 1. To compare the performance, the following root mean square error (RMSE) is used as a metric:

(22) $$\begin{array}{ccc} RMSE(x,y) & = & \left(\sqrt{\frac{\sum_{k=1}^{N}{\left(x_{k}-{\hat{x}}_{k}\right)}^{2}}{N}},\sqrt{\frac{\sum_{k=1}^{N}{\left(y_{k}-{\hat{y}}_{k}\right)}^{2}}{N}}\right), \end{array}$$

where $x_{k}$ and $y_{k}$ are the true position values, ${\hat{x}}_{k}$ and ${\hat{y}}_{k}$ are the estimated position values at time *k*, and *N* is the total number of estimates.

Table 2, Table 3 and Table 4 show the results of the Monte Carlo simulations. Here, each entry denotes the RMSE in *x* and *y* positions as (RMSE(x),RMSE(y)). As listed in Table 2, all algorithms perform similarly with a maximum error of the order of $10^{-1}$ mm in the presence of white noise-affected measurements. In the presence of isolated and simultaneous disturbances, however, PA-EKF and SA-EKF perform much better compared to EKF as the RMSE values decrease consistently. Similar trends are observed in the linear case (Table 3), where the RMSE reduction is of the order approximately 4 mm between EKF and SA-EKF. One can also observe that SA-EKF performs better compared to PA-EKF as in the changing disturbance conditions, SA-EKF adapts faster at each time step as compared to PA-EKF which waits for a complete batch of measurements before updating the measurement noise covariance matrix. In the nonlinear case (Table 4), similar observations can be made that, in the presence of white noise, all three approaches perform with similar RMSE values, whereas, for the isolated and the simultaneous disturbance cases, PA-EKF performs better than EKF, and SA-EKF outperforms both of them as evident by the reduction in RMSEs.

## 6. Conclusions

In this work, two adaptive approaches for EKF were proposed that update the measurement noise covariance matrix based on the past measurements. The first approach, PA-EKF, updates the measurement noise covariance matrix once a batch of measurements is obtained. After that, it waits for $N_{p}$ measurements to update the covariance matrix. SA-EKF works similar to PA-EKF, as it first waits for $N_{s}$ measurements and sequentially updates the batch as new measurements are obtained. This way, SA-EKF updates the measurement noise covariance matrix after each new measurement, while maintaining $N_{s}$ measurements in the batch, whereas PA-EKF updates the measurement after each batch of $N_{p}$ measurements. These approaches were directly incorporated within the EKF algorithm and were validated through static, linear, and nonlinear scenarios in the presence of three types of disturbances: nominal, isolated, and simultaneous. As a result, it is shown that both the proposed approaches have provided better state estimation performance compared to EKF in all the scenarios that were considered. It can be seen from the results that both PA-EKF and SA-EKF are more robust and can adapt to an environment with ever-changing disturbances caused by factors like weather, obstacles, etc. To validate the accuracy of the results, Monte Carlo simulations were conducted with 1000 simulations of all approaches with the tag under static, linear, and nonlinear motion in all three disturbance environments (white noise, isolated disturbance, and simultaneous disturbance). To compare the results, the root mean square error was used as a metric. It can be seen from the results that the proposed approaches offer an improvement in the performance of localization of the UWB sensors. Furthermore, the proposed approaches provide an option to engineers to select a proper algorithm based on a trade-off between computational burden and accuracy. Therefore, further research will be conducted to combine both approaches into a single approach to make the most of the advantages of each model. Moreover, a study on the noise characteristics, especially the noise amplification factor, will be conducted to understand and improve the model based on actual sensor behavior in the presence of sensor disturbance or interference caused by obstacles. In the future, a methodology may be devised to obtain an optimal batch size for both methods.

## Figures and Tables

**Table 1:** Simulation parameters.

Description	Unit	Value
Measurement Update Rate	Hz	10
Simulation Final Time	s	300
Process Noise ($\sigma_{p}$)	m	$10^{-4}$
Nominal Measurement Noise ($\sigma_{m}$)	m	0.04
Noise Amplification Factor (η)	−	4.0
Anchor 1 Position	m	(0, 0)
Anchor 2 Position	m	(20, 0)
Anchor 3 Position	m	(10, 17.3205)
PA Batch Size ($N_{p}$)	−	50
SA Batch Size ($N_{s}$)	−	50

**Table 2:** Monte Carlo simulations for static case (all values in mm).

	White Noise	Isolated Disturbance	Simultaneous Disturbance
EKF	(1.76, 1.76)	(2.77, 2.74)	(4.13, 4.00)
PA-EKF	(1.83, 1.82)	(2.20, 2.18)	(2.85, 2.64)
SA-EKF	(1.83, 1.82)	(1.91, 1.88)	(2.45, 2.33)

**Table 3:** Monte Carlo simulations for linear case (all values in mm).

	White Noise	Isolated Disturbance	Simultaneous Disturbance
EKF	(5.26, 5.48)	(10.27, 9.05)	(13.62, 13.24)
PA-EKF	(5.37, 5.59)	(7.27, 6.89)	(11.75, 11.08)
SA-EKF	(5.37, 5.61)	(6.40, 6.20)	(10.83, 10.03)

**Table 4:** Monte Carlo simulations for nonlinear case (all values in mm).

	White Noise	Isolated Disturbance	Simultaneous Disturbance
EKF	(2.52, 2.81)	(3.74, 3.79)	(7.38, 6.35)
PA-EKF	(2.59, 2.89)	(2.89, 2.92)	(5.64, 5.17)
SA-EKF	(2.59, 2.89)	(2.64, 2.72)	(5.03, 4.50)

## Data Availability

The data presented in this study are available upon request from the corresponding author.

## Abbreviations

The following abbreviations are used in this manuscript:

CKF	curvature Kalman filter
EKF	extended Kalman filter
KF	Kalman filter
LS	least squares
MCU	micro controller unit
PA-EKF	piecewise-adaptive extended Kalman filter
PF	particle filter
PS	positioning system
RMSE	root mean square error
SA-EKF	sequential-adaptive extended Kalman filter
UWB	ultra wide-band

## Appendix A. Further Analysis

The noise amplification factor (η) may vary from low to very high values depending on the obstacle material, shape, and size [21]. This work considers the value of η=4 to compare the performance of the proposed approaches in different environments. η may change in different environments and, therefore, to validate the proposed approaches against the traditional EKF method, this study considers a Monte Carlo simulation of the three techniques applied to a static tag in the presence of isolated and simultaneous disturbance cases as defined in Section 2.2. η is increased from 2 to 7 [21], and the corresponding RMSE performance of the three approaches is compared in Table A1 and Table A2. The results are simulated 100 times to validate the accuracy of the results. Table A1 compares the performance of the three approaches in the presence of isolated disturbances, and Table A2 compares the performances in the presence of simultaneous disturbances.

**Table A1:** Monte Carlo simulations for variation of η in isolated disturbance case (all values in mm).

η	EKF	PA-EKF	SA-EKF
2	(2.09,2.00)	(2.02, 1.88)	(1.99, 1.91)
3	(2.41, 2.36)	(2.10, 2.05)	(1.93, 1.91)
4	(2.82, 2.86)	(2.18, 2.35)	(1.96, 2.01)
5	(3.43, 3.33)	(2.44, 2.54)	(1.85, 2.01)
6	(3.71, 3.69)	(2.61, 2.64)	(1.96, 2.02)
7	(4.42, 4.27)	(2.96, 2.91)	(1.99, 1.95)

In Table A1, it can be observed that when η is low, the difference in the performance of the three approaches is much less. However, when η is increased from 2 to 7, the performance of EKF reduces as evident through the increase in the RMSE. Similarly, the performance of PA-EKF also decreases; however, the RMSE values are less compared to EKF. On the other hand, in SA-EKF, one can observe that the performance remains consistent and the RMSE at η=7 is half of the RMSE of EKF. This highlights the consistent and improved performance compared to EKF.

**Table A2:** Monte Carlo simulations for variation of η in simultaneous disturbance case (all values in mm).

η	EKF	PA-EKF	SA-EKF
2	(2.46, 2.45)	(2.26, 2.21)	(2.16, 2.17)
3	(3.08, 3.21)	(2.51, 2.49)	(2.34, 2.35)
4	(4.02, 3.93)	(2.86, 2.58)	(2.45, 2.30)
5	(5.05, 4.90)	(3.48, 2.93)	(2.77, 2.44)
6	(6.16, 5.81)	(3.74, 3.21)	(2.72, 2.50)
7	(7.13, 6.81)	(3.89, 3.64)	(2.73, 2.43)

In Table A2, similar trends can be observed, as in the case of EKF, the RMSE increases from 2.46 to 7.13 mm in the *x*–position. However, in the case of PA-EKF, it only increases up to 3.89 mm and in the case of SA-EKF, it remains under 3 mm throughout the variation of η.
